# Medical Etiquette : Or an Essay on the Laws and Regulations, Which Ought to Govern the Conduct of Members of the Medical Profession Towards Each Other

**Published:** 1840-01-01

**Authors:** 


MEDICAL ETIQUETTE; OR AN ESSAY UPON THE LAWS AND REGULATIONS, WHICH OUGHT TO GOVERN THE CONDUCT OF MEMBERS OF THE MEDICAL PROFESSION IN THEIR RELATION TO EACH OTHER. Compiled exclusively for the Profession. By *Abraham Banks*, Esq. Member of the Royal College of Surgeons, London; Licentiate of the Company of Apothecaries; and late Surgeon in the Hon. East India Company's Service. Better perhaps expressed thus: M.R.C.S.L.; L.A.C.L.; S.H.E.I.C.S.; &c. &c. London, Charles Fox, 1839.

MR. BANKS is certainly a humourist. The abbreviated titles are a *capital* joke. And he is an enthusiast in the cause of medical etiquette. He does not mince matters—he is no milk and water, juste-milieu man—he throws himself body and soul into etiquette, and he comes out professional honour personified.

He tells us that his Treatise—

"Is not addressed to him,* who from accident, or any other cause, having been called in to attend another man's patient, endeavours by every mean and underhand insinuation to wrest that patient from the original attendant; nor is it addressed to him, who may have been sent for, whilst another was in attendance, and taking advantage of the absence of that other, after much apparently skilful examination, and many enquiries to no purport whatever, casts up his eyes to Heaven, and with that mysterious waving to and fro of the hands, and that significant medical '*hum*,' exclaims, aghast in wonder and amaze, 'What a pity you had not sent for *me* before—if I had only been called in *six hours* sooner —but it is now too late, it has gone too far;'—nor to him, who under similar circumstances, on being shewn the medicine which had been prescribed, so natural to the friends of the patient, examines the devoted bottle with wonderful sagacity, and after due smelling, and sniffing, and tasting, and various other mountebank operations, dooms the unconscious deadly potion, consisting perhaps of a little saline mixture, to the awful punishment of ejection from the window;—nor is it addressed to him, who resolves to build up a reputation for fame at any price, who depends more for success upon detraction, and sapping the reputation of others, than on any intrinsic merit of his own. These generally consist of the out-pourings of the schools, of novices who have only seen the A B C of practice, and possess a little smattering of the science, but who, as they gain experience and knowledge, outgrow this absurdity. We should hope the maturer part of the profession was a stranger to such glaring outrages as these; if, however,

* "All the imperfections of character alluded to are unfortunately taken from living practitioners."

there be any of advanced practice capable of such conduct, all that we can say is, that they are too contemptible even for castigation." xii.

We wish we could say that we thought Mr. Banks quite right in this aspiration. We are afraid that it is not the out-pourings of the schools only who practice these mean arts. Some men of more years and of greater standing know the trick and play it. But the press, like Ithuriel's spear, *may* shame or frighten them, and never was a subject more legitimately suited to its lash than such paltry and dishonourable conduct.

"But," continues Mr. Banks, "to aim at higher game, it is not addressed to him who takes advantage of having once been summoned to a family, to call again unasked, and by sundry intimations endeavours to lower the estimation in which the regular attendant is held, and thereby pave the way for his own admission; to call such conduct unprofessional and dishonest, is not rendering it full justice; it is base and unmanly in the extreme; it is assassination in the dark—the resort of the coward. The man who gives his adversary due notice of attack, and thus enables him to withstand the shock of his charge, who openly declares him to be an ignorant blockhead, and unfit to practise his profession, is a noble and honest character compared with this other. Nor, lastly, is it addressed to the man who carries his profession upon his back wherever he goes, who never loses an opportunity of instilling into the minds of all those who have the misfortune to pass before him, that he is the incomparable; that if a person really wishes to be cured, to *him* they must go; that of all the professors of medicine he is, emphatically, *the professor*—the nonsuch of the profession; or, to him who seizes the opportunity, when the family are present, of reprimanding a young practitioner for alleged indiscretion, for the malicious purpose of injuring his reputation, or who expatiates in his absence on the advantages of employing a physician exclusively, who understands disease and infirmities better, and who, when unhampered by a general practitioner, orders little or no medicine: for such men there are, to their shame be it said, amongst the leading physicians of the day." xiv.

We would fain hope, nay we do believe, that the last imputation is unjust. There is no "leading" physician nor consulting surgeon who could so behave to "practitioner," old or young. We cannot credit it.

Mr. Banks' essay *is* addressed to the honourable men (the vast majority) among us. Even such a man, with the finest self-respect, the nicest sense of decorum, the purest spirit of honour may be placed in embarrassing situations. For instance, says Mr. Banks, he may be sent for in a hurry when the regular attendant happens to be out of the way; he goes and prescribes, then of course retires, leaving the patient under the care of the regular attendant; but as he is going, the family request him to call again; he pleads professional honour and etiquette, proffering his services at any time under similar circumstances; the family still urge him to continue, asserting that it will make no difference so far as the previous attendant is concerned, as if he refuses, they will send for some other person; a man, whose feelings are not over keen, has no difficulty here. But we trust the profession embraces thousands of members, who would feel it a very unenviable situation.

We shall not follow Mr. Banks through all the questions that he solves, or tries to solve. We shall content ourselves with discussing some of the more knotty points of honour.

1. *When one Practitioner attends a Case of Midwifery, or an Accident, for another.*

" There seems to be no general understanding in the profession, as to what is to be done in respect to the fee; some will take it, and keep it, and occasionally the patient also if they can; others compromise the matter, and retain a portion; whilst others again persist in refusing any consideration whatever. It is perhaps more congenial with the highest notions of honour, and more consistent with true dignity, to adopt this last practice, but many inconveniences necessarily arise from it; for instance, unless it be universally followed, it leads to a great deal of unfairness. A very sensitive practitioner may always persist in refusing any remuneration, whilst he under similar circumstances shall have every fee wrested from him: this is most unjust, and places an honourable man in a very disadvantageous position. To give another illustration of its injustice —there may be two men in a neighbourhood, the one having a very large, and the other a small practice; the chances in this case will be, that the man of small practice will do ten, or more times as much for the other practitioner, as that other does for him, so that he becomes in a measure his assistant, without the fair compensation. Again, in London, from the inconvenient practice of employing medical men at such great distances, it often happens that about the outskirts, or in the suburbs, a man may have to attend more cases for others than for himself; here also he acts the part of assistant to the more established practitioner.

Now, to obviate these difficulties, some persons have proposed to divide the fee; and amongst many this is done by a previous understanding to that effect, and if all fees were of much the same magnitude there could be no objection urged against this arrangement; but as some practitioners attend for almost as few shillings as others do for guineas, it seems unfair that he who may have paid two or three guineas should in his turn only receive a crown, or less, for conferring a similar kindness. The inconvenience also of having a fixed remuneration must be clear to every one, as in many cases a practitioner might have to pay more than he receives.

To remove all these difficulties, we would therefore propose that, whenever one practitioner is accidentally called in to attend a labour for another—excepting amongst intimate friends, who of course will consult their feelings much better, by allowing a larger latitude of generosity, if we may use that word—he shall receive half the fee, recognizing no fee below one guinea, or above two, and that this shall in future be considered perfectly compatible with the highest notions of honour and professional etiquette, as it would appear to be consistent with equity and common sense. The same rule, with some slight modification, might apply to accidents, especially those of a serious character." 3.

This proposition seems to us to be fair enough. We observe that some of our contemporaries scout the idea of any such arrangement between general practitioners, no such being adopted by *Pures*. But the cases do not seem to us to be quite analogous. General practitioners live more by a limited number of patients in a given range, and by connexion, than the Pures do: and a more strict code of reciprocity becomes requisite. There is not so much danger of the Pures clashing as of general practitioners clashing, and every fence against what might excite bad blood should be erected. It would be well were there a more systematic establishment of local societies among the general practitioners than there is. Many *trespasses* would be prevented.

### 2. *When Two Practitioners meet in Consultation.*

"It often happens, at the desire of the patient, or his friends, another general practitioner is called in, by way of consultation: now there can be no objection to this, nor do we see why two general practitioners should not meet in perfect harmony upon a case, if there were some general understanding in the profession as to the conditions upon which that meeting should take place. As it is now, this often leads to some unpleasantness, and commonly to reluctance on both sides; it usually terminates in one of the parties giving his attendance gratuitously, though we confess we cannot perceive why he should do so any more than a physician, or pure surgeon; it is lending his sanction to a practice, which it is both his interest and his duty to endeavour to overthrow, nor indeed is it fair towards himself. Occasionally he receives the customary fee; this again is unfair towards the other practitioner: for why should two men holding the same rank, possessing the same legal qualifications, the actual experience and knowledge pretty equally balanced, be placed in such different positions? Yet the profession may daily witness the monstrous absurdity of two men of equal grade, the one receiving his guinea, the other his half-crown, for the same services, with this difference, that he who receives the half-crown has to provide medicine in the bargain. Now to obviate this anomaly, we would suggest that when two practitioners, possessing the same *legal* qualifications (for however absurd these may be as the line is now drawn, yet they form the only criterion for us to act upon), meet upon a case, they should be received on precisely the same footing, and each have the same amount of remuneration, and at *the same time*, for that particular consultation, whether this be a guinea, a half-guinea, or a crown." 7.

No doubt, this would be best; the difficulty lies with the patients. They may object to an additional charge on the part of the attendant practitioner—and it would seem odd for the other to receive only the usual sum for the daily visit. It is clearly impossible to lay down a fixed rule, when the caprices of patients interfere with its operation. If the ordinary attendant stands too much on a point he may offend. *There* is the danger. We would suggest that unless both practitioners can receive at least half a guinea each, the consultant should make no charge. He must place, of course, his gratuitous visit, not on the ground of friendship to the family, but of professional etiquette.

### 3. *Affectation of Mystery.*

"We have heard physicians use such words as 'secundùm artem, ad deliquium, toastùm boastum,' &c. &c., when talking to a general practitioner before others, such can only impose upon the ignorant, and cannot fail to lower a man in his own estimation; the profession should be too proud to violate the laws of good taste and honesty, to bend to popular error. We remember some time since dining at a house where there was a lawyer at table, who, amongst many other professional terms, said, nothing was so mean as a *tu quoque;* if this gentleman could have seen how ridiculous he appeared, his countenance would doubtless have betrayed some embarrassment of the internal man.

When any person unnecessarily uses technical terms in the presence of others who may not be supposed to understand them, we regard it as a direct insult to those persons; it is in fact laughing at them. Closely allied to this habit, is that of clothing medicinal preparations in false colours, such as mixing rose pink with linseed meal, vermilion with epsom salts, burnt sugar with goulard water, &c. &c. We know that strong excuses may be pleaded in extenuation; but we may be permitted to deplore that constitution of society, which renders such conduct almost necessary; we believe it to be perfectly incompatible with an ardent love of truth, and a glowing admiration of rectitude.

Many other practices would seem to come very properly under this head, all which, being part and parcel of a system of deception, violate more or less the sacred sanctuary of truth. It is scarcely necessary to allude to that thoroughly beaten path, which has now become nearly obsolete from its palpable nature; such as being called out of churches during divine service, and other public places, and so contriving matters as to be riding hard by, when people are coming out, his horse foaming and sweating—poor animal! all in the cause of falsehood. But this has given way to other arts equally reprehensible, though of a more refined character, and not quite so obvious to public perception; such as singing very loudly over and above all the rest of the congregation, taking a conspicuous pew, and sometimes mounting on a hassock, in order to be well seen; giving the responses in very audible language, so as to excite the observation, 'Who is that pious gentleman?' making himself very officious, particularly in the charitable department, so far as the collecting goes, more especially if there is any chance of filling a medical appointment. A petition for a charity forms an excellent plea for calling on the wealthy, and putting in a good word for number One—the more so, if nobody else will do it; bowing to every one he meets, though, perhaps, he has never seen the person before; assuming a very religious tone according to the character he has to deal with, as, 'Well, Ma'am, we have maturely considered your dear little girl, and ordered such and such medicine, which, by the blessing of God, we hope will have the desired effect:' all this hypocritical cant, if it be not criminal, is truly disgusting.

Another recent manœuvre, which is sometimes practised, is putting up counterfeit medicines, and letting them lie about the counter in the surgery or shop, so as to give a false impression of business; talking largely, and contriving, if any excuse can possibly be obtained for so doing, to introduce the name of some nobleman or baronet into all his discourse, chiefly before strangers. We have witnessed instances where some unfortunate peer, who may have accidentally got his name upon an apothecary's books, has had that name mangled most unmercifully, as, 'John, has my Lord such a one had his medicine? be good enough to send that medicine to his Lordship directly; I will attend to you, Sir, as soon as I have ordered something for my Lord ——— ' &c. We remember hearing of a man who could not open his mouth without letting people know that he kept a horse and chaise; a bet was made upon the strength of this, that he could not answer the simplest question without introducing these essentials of his establishment. The question put was direct enough; he was asked what o'clock it was? and answered, 'When I, with my wife, passed the Horse Guards this morning in my horse and chay, it wanted,' &c." 14.

Mr. Banks has a hit at sending medicines to the wrong houses, "advice gratis," &c., but the Pickwick Papers have immortalised the "Sawyer, late Nockemorfs," already. We fear that our Professor of Etiquette is a *little* Utopian.

"Not quite so palpable," says he, "as these is that system of being all things to all men; of studiously avoiding expressing a sentiment which may be thought not exactly to correspond with another's; of feeding the ruling passions of others, whatever may be their tendency; of holding no opinion on general or public subjects; of adopting a reservedness in all mixed society. All this may be very good policy, but there is something servile in suppressing one's sentiments, lest they should not harmonize with those of other persons; in sounding one's way with such cautious timidity, lest a rumour should get afloat that one is not of the orthodox: but, above all, would we deprecate, in the strongest terms language is capable of affording, that violation of principle in public matters which sacrifices conscience to success; a crime than which we know none greater, so utterly subversive of all the rights of society. The man, endowed with the privilege, and who goes not to the poll, is guilty of a most flagrant dereliction of duty: but he who goes and votes in violation of his conscience is such a monster of iniquity, branded so imperishably with the marks of infamy and disgrace, as ought for ever to exclude him from the pale of civilized life, and render him

'A fixed figure for the hand of scorn,
To point her slow unmoving finger at.'" 16.

This is very well, but doctors must, after all, "please to live." We are so dependent not merely on the patronage, but even on the whims and caprices of the public, that few can afford to shew independence. We do not agree with Mr. Banks on the necessity for avowing political opinions. As a profession we should rather hold aloof from general politics. Whig, Tory, Radical, Socialist are all our patients, and, as we must necessarily come into immediate contact with all, our intercourse should not be embittered, nor the benefits of medical science neutralised, by the gall of political rancour. No medical man need become a partizan of any of the factions that deform the state. We would not have it supposed that we advocate sycophantic subserviency. Far from it. No man need palter with the prejudices or the passions of his patient. He should hold himself aloof from both, and display that reserve which is the attribute both of prudence and philosophy.

### 4. *Mode of Payment.*

Mr. Banks has as great a horror of a bill, as any patient can have. He prefers fees, and, on "the bird in the hand is worth two in the bush" principle, as well as on every other, there can be no doubt of their superiority. He would go even as low as a shilling, and a half crown would be more than twice as good. Amongst other reasons, he esteems it because it would give patients a practical lesson of providence. There cannot be a doubt of the superiority of the plan of being paid by fees. But here again the patients are the "obstructives." The old method has become too deeply rooted, to be easily plucked up. Yet there are practitioners who have adopted the new, and with complete success. We are inclined to believe that many of the younger men will make the experiment.

Mr. Banks touches on another subject.

"The plan of present payment, too, would remove all that doubt which exists as to paying for domestics, which is often the source of much annoyance, and, occasionally, of legal dispute. There is a good deal of difference in the practice of persons respecting this; but from some experience in these matters, we hesitate not to say, that all respectable people do it generally; and, in equity, it would seem that a domestic, who has been some time in a family, has a moral claim, when it can be afforded without inconvenience. It is most unjust, and even inhumane, to throw a servant upon her own resources in case of illness, who may have faithfully discharged her duties for several years; yet we have been witness to an instance where the master refused to pay the medical expenses of a servant under such circumstances, though attended in his house both by a physician and apothecary; a man of considerable property, living in Brunswick-square, and the head partner of one of the largest legal firms in London. This is quite the exception to what is customary; and in all cases, where the master or mistress employs the medical attendant, or is aware of the attendance, we think they ought to be responsible, unless they have previously intimated to the contrary; and that it rests with them to state so, and not with the practitioner to make the inquiry." 22.

The physician and consulting surgeon is much less frequently remunerated for attendance on a domestic than the general practitioner. This is one of the cases which must be left to the determination of circumstances, and to the discretion of the medical attendant. Nothing can be more impolitic than to press a claim which is reluctantly allowed. Wealthy and liberal persons may pay for their servants—persons who are neither liberal nor wealthy will not; and if pressed to do so may *bolt*. It is a ticklish thing for doctors to stand too much upon right.

5. *Are medical Pupils to pay?*

We should decidedly say, no! And yet we have known a London surgeon in large practice and of scientific eminence take fees from his own pupil, knowing him to be such. This was too bad.

6. *Are Medical Men to pay one another?*

Undoubtedly not. Of course one practitioner is not bound to find medicine for another; he cannot be expected to be *out of pocket*. But his time (within reasonable limits), and his skill (provided the tax on it is not excessive), should be at the service of his sick brother. To this there must, in the nature of things, be some limitation. A man in large practice in London can scarcely be expected to go such a distance in the country as will cost him a day or two, without such a remuneration as will prevent his being a loser. A great difference should be made in the *amount* of a fee paid, under these circumstances, by a professional or a non-professional person, and, perhaps, something should be presented in the lieu of money. But for a man to go unremunerated altogether would be both unjust and impolitic. We say impolitic, for men in good practice would find or make excuses for their non-attendance, if they were served so.

" The same regulations, which apply to medical men, we think, should apply whilst they are living to their families; but not after their decease. That liberality which dispenses indiscriminately its favours upon all—the rich and the poor, the deserving and the undeserving, we strongly condemn, which often extends even to unprofessional acquaintances; we call it a wanton, wasteful, irrational, and mischievous liberality. The practice of some persons, when they happen to have a medical man at their table, of pumping him, and so getting back the value of their dinner, is mean and paltry in the extreme: and the claims which relatives often put forth, are very unreasonable and unfriendly; instead of encouraging a young professional person, they only serve to prey upon him.

With respect to the families of medical men after their decease, we do not see what other claim they possess to be placed on a different footing with the rest of the world, than the rights of friendship, and the plea of straitened circumstances. The notion entertained by some that the relations of deceased medical men, without reference to state, possess a decided claim upon all the members of the profession, seems to us an absurd, unjust, and visionary idea. A little extension of the same principle would produce a community of property, and lead all persons to throw their labour, and goods, into the general weal; which, however disinterested it might be, we do not think would tend to advance the civilization and improvement of mankind. A habit of grasping is by no means a general failing in the profession; the fault lies a little the other way; and there are very many who much prefer indulging their liberality, than extending the lining of their pockets. These remarks have been occasioned by a circumstance which came under our observation some time since, where a young practitioner had been attending upon another much older, and in very superior circumstances, most assiduously, and so far as thanks and verbal acknowledgments went, was amply repaid; but no pecuniary consideration was offered, although the attendance had been long, and much medicine, and many things unconnected with medicine, which are usually kept in a chemist's shop, had been supplied; the parties never having heard of each other before the accidental occurrence which bronght them together. To suffer this to pass by unnoticed in any other way than words, we think was both illiberal and undignified." 30.

We think so too. He must be a shabby fellow who does not send his brother practitioner a piece of plate at least, when under such an obligation.

7. *Remuneration for Prescriptions and Medical Certificates.*

" It has always appeared to us most unreasonable on the part of the public to request, and very injudicious also on the part of medical men to give up, a copy of one of their own prescriptions which has proved extremely serviceable, without equivalent; yet it is a common occurrence for any patients, on the plea of going into the country, to request of their medical attendant a prescription of some medicine they may have been taking a considerable time, and may possibly continue to take for years.

From the foolish system of charging for attendance under the form of medicine, the general practitioner has probably been receiving three shillings for that which would be prepared at a chemist's for half, or less; of course that prescription never comes back again; and thus a medical man gives up, perhaps, a small annuity, for no consideration! However, no one with the least pretension to liberality, could refuse a patient's making the request under such plausible circumstances, though we have full reason to believe that it is often done for the very purpose of getting it put up cheaper. A bold demeanour is not the general characteristic of the profession, and thus the majority concede to the application, though usually with reluctance; some refuse altogether upon the absurd plea that it cannot be written out, or that no other person could prepare it, and offer to supply a sufficiency of medicine for the time of absence. This would seem to be very fair, as every man is certainly entitled to the benefit of his own preparations, though it would be much more independent to state the real reason of refusing. But this cannot always be done; patients may be going abroad, or to leave the place entirely, and medicine will not invariably keep, and much inconvenience will sometimes arise from carrying or sending it to distances: now why a prescription under such circumstances should not be paid for, we cannot tell, except from the absurdity of custom." 32.

Mr. Banks suggests that where a patient applies for a prescription of a general practitioner of medicine, which has proved its value, on the plea of going into the country, or any other pretext whatever, a fair consideration be demanded as a matter of course, proportioned to the circumstances of the patient, and the probable use it is likely to prove of, the minimum amount being equal to a physician's fee; and to prevent individual prejudice, that this be universally adopted, and that the profession manifest a becoming spirit when the public shew any disposition to impute improper motives to this regulation. Something of this sort should certainly be generally agreed on amongst general practitioners.

Mr. Banks greatly dislikes the idea of writing certificates, and things of that sort, without pay. The thing may be unpleasant, but it is not easy at all times to avoid it. The request is made, in the majority of instances, by patients whom we have been attending, and it would appear invidious to demand, on all occasions, a remuneration. What Mr. Banks says of law is very true. Nothing is done in it for nothing. But the fixed fees of lawyers and the fluctuating charges of doctors do not admit of strict comparison. The mode of doing business in the two professions is quite different, and so are all the circumstances. No doubt, there is too facile a temper with regard to fees on the part of medical men. The affluent reject them, and the more needy members of the profession suffer. A patient says:—" Oh, Mr. so and so, (a physician or consulting surgeon) would take no fee, and this medical man makes a charge." Until the profession agree on these matters we see no prospect of any satisfactory arrangement. If a practitioner is too *exigeant* he *will* suffer, and if forced to be too liberal he *does* suffer.

"There are certificates of death in consequence of the new law, which give a great deal of trouble. Undertakers, sextons, and clergymen, all receive their fees before they will stir an inch, then why should not a medical man receive his, without whose authority none of them can legally act at all? Is it anything uncommon for a clergyman to refuse performing the burial-service before he has received his legal rights? And yet the poor medical practitioner, who has had all the responsibility of the illness, and perhaps never received a farthing for his trouble, is expected to write these certificates without any acknowledgment! All this is utterly monstrous, and part and parcel of the remnants of the old system of demanding medical evidence at inquests gratuitously. We respectfully refer it to the whole profession whether, for the future, it would not be more consistent with justice, and more calculated to support the respectability of the profession, to make an adequate charge, for looking over, or signing any paper, certificate, or document whatever." 37.

This recommendation will not, assuredly, be universally acted on. It were much to be wished, however, that some understanding could be come to on the part of the profession. As matters stand all suffer, but more particularly the general practitioner. Not that he has more to do gratuitously, in this way, than others, but he can, generally, less afford these irruptions on his income, than his more wealthy friends, the *pures*.

8. *When a Practitioner is accidentally sent for to the Patient of Another.*

When this is really urged with truth and sincerity, and not out of complaisance, we cannot see that any stretch of honour requires a man to forego attendance, because this would lead to a most overbearing, tyranical custom; it would, in fact, be tantamount to saying to the public, that whatever practitioner you choose to employ in the first instance, him you shall continue to employ: this would be an insufferable dictatorial measure. The public have an undoubted right to send for whom they choose; and if they feel dissatisfied with one man they are privileged to send for another; and no reflection, under these circumstances, can equitably be cast upon a practitioner, provided he has done nothing directly or indirectly to bring the former one into disrepute. But here we feel particularly anxious not to be misunderstood; for our own part, we should be very reluctant to obtain a patient this way; much suspicion will always attach to such a case; nor do we consider that a patient, so acquired, will ever reflect much credit on the possessor. Persons are often capricious, and feel mortified if a man has happened to be out of the way once or twice; and some trifling excuse made for him will mostly rectify matters, procure the apologist the esteem of both parties, and more especially the sanction of his own conscience (we shall, perhaps, be thought to be wandering in the wilds of a visionary Elysium; but we would reiterate, that we are only writing to men endowed with the moral qualifications of practice); and at any future time, if he happens to be sent for first, he unhesitatingly goes without the possibility of the most distant reflection being cast upon his character; but by construing the dissatisfaction expressed, which may only be temporary to his own advantage, he most justly lays himself open to the reception of similar treatment from others." 45.

It is quite clear that a man has a right to dismiss his medical attendant if he chooses. That being the case it would be contrary to reason for another practitioner to refuse to attend. The absurdity as well as wrong to the patient would be monstrous. But if a man is accidentally called in the place of another, the case wears a different aspect. The man who endeavours to filch his neighbour's patient *then* is a rogue. Manœuvring and intrigue are too often resorted to, and the

"I would not, waits upon I would,"

is too common and too palpable. The interests of the profession require that such conduct should be stigmatized, and the strictest code of professional morality enforced. The arm of opinion cannot reach all offenders, but it will deter many from wrong and it will intimidate most.

We said that Mr. Banks was a bit of an enthusiast. He has a very high notion of doctors. He proposes that we should turn conservators of the public morals; a whole profession of censors!

"We would now appeal, with all that solemnity which the importance of the thing demands, whether the members of the medical profession would not do well to take under their protecting care the glimmering embers of morality; for if they do not, who shall? Should the Law make the attempt, it would be like nursing a serpent at her very bosom; for moral law and statute law are at total variance; the one of a high, noble, refined, and elevating character; the other of a low, mean, grovelling, and outreaching nature, the demoralizing tendency of which is but too apparent in the majority of its professors. The Church! But when has Sectarianism shewn herself favourable to the development of mind? When has she proved herself capable of taking those large and extended views of human nature, which comprehend the whole race? Church history has hitherto been little else than one continued series of oppressions, of prejudices, of tyrannies, of usurpations, of the blackest description. We fear that the mild and delicate Plant of morality would wither under the protection of Sectarianism, which, however admirably adapted for sowing the seeds of dissension in families, and fanning the flame of civil discord, is wholly unfitted for this nobler function. The Educator! the natural Ally of morality, to whom society would look as to a parent for assistance—but what has he as yet done, but train some for oppression, some to oppress? From the contemptible remuneration, and the low estimation, or rather the suspicious aversion, with which he is generally viewed, his profession has become merely the refuge of the mean-spirited and the destitute, where vanity, jealousy, and dogmatism are the prevailing passions. Alas! Thou poor Morality! If thou art deserted, and forsaken by the only men capable of throwing a shield of protection around thee, and infusing new vigour into thy spirit, the members of the medical profession, what hope hast thou? Nay; despair, and die!" 53.

If this be so, we fear that morality is in a very asthenic state. But what will the Church say to this? Ne sutor ultra crepidam, no doubt; "stick to the body, to the phthisic and the physic" quoth the parsons, "but leave the soul to us." And we really think the Cloth would be hardly dealt with if we served them in the fashion Mr. Banks would wish. Besides, the Church has the munitions de guerre, fifty millions of revenue, as Lord John remarked. If that will not *force* morality, we are afraid that no medical manure will answer.

9. *When a Second Party is called in to decide upon the Treatment of Another.*

"Nothing can be more agreeable to a rightly constituted liberal mind, than to give an opinion in confirmation of the judicious treatment adopted by another; but when it happens that there has been most palpable neglect, or most decided maltreatment, a conscientious man is necessarily thrown into a very unenviable moral dilemma. Strongly reluctant to infringe in the least degree upon the claims of generosity, or to lay himself open to the slightest imputation of illiberality, he yet feels a moral responsibility resting upon him to give his honest opinion; what is he to do? He must apply for assistance to Moral Philosophy; she will teach him that truth constitutes the great bond of union between man and man; and that however adverse to his feelings, yet his duty to society, his duty to himself, his duty to the individual soliciting his opinion, call upon him to declare his real sentiments.

It has appeared to us that a very common notion which has gone abroad, that under all circumstances the previous treatment of a patient is to be supported and confirmed, has its origin in a very false refinement of feeling; and when duty and feeling clash, we apprehend there will be few who will hesitate an instant which is to be paramount. When it is considered how differently the time which ought to be devoted to the acquisition of knowledge and experience, is passed by different individuals, it is not fair that the man who has devoted his whole time, morning, noon, and night, to his profession; who has been an unceasing attendant in the dissecting-room; who has sat for months over offensive subjects, until their revolting appearance through habit had almost become attractive; who, whilst his hands have been employed in laying open the works of nature, has thrown the whole force of his mind upon the subject to explore her more recondite operations; who has let no opportunity slip of collecting new facts, and arranging those in his possession; it is not fair that such a man should have no advantage over him, whose whole thoughts have been centred in most opposite things, to whom the Theatre, the Ball-room, and the Card-party, have presented more alluring attractions than the Chamber of sickness, or the Portals of Death." 56.

It appears to us that, on many accounts, the rule should be to approve, or, at all events, not to disapprove of previous treatment. Judgment is fallible, medicine difficult, symptoms deceptive, and every allowance should be made for mistakes. We all make them in turn, and no man has a *right* to be censorious. There may be extremely gross cases, where common justice and common decency require reprobation of what has been done. But *honest* error should not be exposed, and the interest both of science and the sick, to say nothing of charity, demand that confidence in the medical attendant should not be destroyed. That is (we may be wrong) our opinion, and on such we should ever act, as we would wish others so to act towards us.

Here we must conclude, recommending Mr. Banks's little book for its high tone of honour, and rigid cast of morality. It would be well for the Profession were its precepts laid to heart, and strictly acted on.

